# Recombinant FSH Compared to Clomiphene Citrate as the First-Line in Ovulation Induction in Polycystic Ovary Syndrome Using Newly Designed Pens: A Randomized Controlled Trial 

**Published:** 2016-03

**Authors:** Batool Hossein-Rashidi, Bahareh Khandzad, Ensieh Shahrokh-Tehraninejad, Maryam Bagheri, Mansoureh Gorginzadeh

**Affiliations:** 1 Reproductive Health Research Center, Tehran University of Medical Sciences, Tehran, Iran; 2 Tehran University of Medical Sciences, Tehran, Iran

**Keywords:** Polycystic Ovary Syndrome, Clomiphene Citrate, Recombinant FSH, Ovulation Induction

## Abstract

**Objective:** Since there is still controversy regarding the best first-line choice for ovulation induction (OI) other than clomiphene citrate (CC) in infertile women diagnosed with polycystic ovary syndrome (PCOS), the aim of the present study was to compare recombinant human FSH with CC as the first course of OI in these women.

**Materials and methods:** In this pilot randomized controlled trial, 104 infertile women diagnosed with PCOS were randomized in two groups to receive either CC with the dose of 100mg per day from day 3 of a spontaneous or progestin-induced menstruation for 5 days or rFSH with the starting dose of 50 IU daily {and weekly dose increment of as low as 12.5 IU}, on the day4 of the cycle. They were assessed during a single OI course. The pregnancy rate (PR) and live birth rate (LBR) were the primary outcomes. The follicular response, endometrial thickness, cancellation of the cycles and ovarian hyper stimulation (OHSS) rate were the secondary outcomes.

**Results:** Analyzing data of 96 patients using Chi^2 ^and Fischer’s Exact test (44 in rFSH group and 52 in CC group), both PR and LBR were comparable in the two groups {13.6% vs. 9.6% and 11.4% vs. 9.6% respectively}, with the difference not to be significant (p > 0.05). No cases of OHSS or multiple gestations happened during the treatment course.

**Conclusion:** It seems that rFSH is as efficacious as CC while not with more complications for the first-line OI in infertile women with PCOS. However, due to the limitations of the present study including the small population and the single cycle of treatment, our results did not come out to prove this and more studies with larger study population are needed to compare the cumulative PR and LBR.

## Introduction

Being the most common endocrine disorder in women, polycystic ovary syndrome (PCOS) is also the leading cause of anovulatory infertility. The higher prevalence of PCOS in Asian countries compared to Western ones on one hand and the higher desire for pregnancy in these countries on the other hand further raise the importance of this issue (1-3).

What is already practiced for the treatment of infertility in women with PCOS is the use of clomiphene citrate (CC) as the first modality, and for those who do not ovulate or conceive after maximum six cycles, second-line therapy including combined treatment with CC and metformin is applied (2, 4, 5). Gonadotropins are used as the third-line strategy (2, 4). Addition of IUI in order to increase the pregnancy rates though accompanied by extra laboratory work, more hospital visits and further costs has also been recommended (7). In those infertile PCOS patients who are not responsive to the previous therapies, IVF is regarded as the last resort (2). CC results in ovulation in 75% of anovulatory women; however, pregnancy rate is regarded to be as low as 36% (6). Anti-estrogenic effects of CC on endometrium and cervical mucus are mainly blamed for this difference (6). Therefore, CC does not seem to be as efficient as beforeand this may be due to the emergence of clomiphene failure and resistance (7-11).

Some disadvantages have rendered gonadotropins as the second-line modality and not the first for ovulation induction (OI) in PCOS. These mainly include multi follicular development and high cancellation rate due to ovarian hyper stimulation syndrome (OHSS) (7). Given the fact that endogenous LH is already elevated in PCOS patients, the highly purified or recombinant FSH preparations are adopted presuming the theoretical advantage of better efficacy as well as lower OHSS rate (12-14). Furthermore, anti-estrogenic effects of CC with its main drawback of suppressed thin endometrium can be eliminated (15). Low dose FSH has been safely and effectively applied for OI in PCOS patients following failure with CC therapy (16-21).

 Until now, two studies have looked into the use of low dose recombinant FSH as the first step for OI in PCOS women (18, 19). In continuation to those trials, this study was designed to investigate the outcomes of recombinant human FSH usage as the first-line treatment of infertility in women with PCOS.

## Materials and methods

This study was a pilot double – blind randomized clinical trial with registration number: 11135181N8 which was performed during a one-year period from March 2013 to June 2014 at the Infertility Clinic of Vali-e-Asr Research Centre known to be a referral infertility center in Tehran. The ethical approval was obtained from the Faculty of Medical Ethics Committee of Tehran University of Medical Sciences.

The H_0_ or the null hypothesis of the study was that rFSH usage as the first-line treatment for the OI in PCOS was not associated with superior outcomes. The H_1_ or the alternative hypothesis on the other hand was that rFSH would be more efficacious than CC for OI in PCOS patients in terms of reproductive outcomes. Due to the novelty of this study in our country, it was regarded to be a pilot study consisting of 60 subjects per each treatment arm evaluated for a single treatment course at the first step. 

 Block randomization method was chosen, i.e. using Stats Direct statistics software, random block size of between 4 to 8 was determined. Allocation proceeded by randomly selecting one of the orderings and assigning the next block of participants to study groups according to the specified sequence. This was done by a third person blind to the study. The investigators themselves were blind to the patients list. A total of 120 women with primary infertility, were enrolled in the study. 

The inclusion criteria were as follows:

     1. The diagnosis of PCOS according to the revised Rotterdam criteria by the European Society for Human Reproduction / American Society of Reproductive Medicine infertility. 2. Age range of 18 to 40 years old. 3. BMI ≤ 30 kg/m2. 

     The exclusion criteria were: 

     1. History of previous infertility treatment. 2. Other infertility problems like endometriosis or male factor infertility. 3. Abnormal uterine cavity or tubes based on hysterosalpingography or laparoscopy. 

Hormone profile including serum FSH, LH, PRL, AMH, estradiol, DHEAS, testosterone and 17OH-progesterone were measured for all the participants. Sixteen subjects were excluded with 12 not meeting the inclusion criteria and 4 refusing to participate. Finally, 104 subjects remained who were randomly assignedto receive either CC or recombinant FSH as their first treatment course.

***Treatment protocol***

In one arm, Clomiphene Citrate (Aboureyhan Co. Tehran, Iran) was given to 52 patients with the starting dose of 100 mg per day from day 3 of a spontaneous or progestin-induced menstruation for five consecutive days. In the other arm recombinant human FSH (Gonal-F, Merck Serono Co. Italy) with the starting dose of 50 IU was prescribed from cycle day 4. Weekly increase of 12.5 IU was done if necessary to induce the follicular response. Transvaginal sonography was done every other day from day 11 until at least one follicle ≥ 17 mm was seen; in that case, ovulation was induced by the administration of 10000 IU of human chorionic gonadotropin (hCG) and intercourse was recommended for the following two days.

Pregnant women underwent an ultrasound at 7 and 11 weeks of gestation. The pregnancies were classified as clinical or ongoing and singleton or multiple according to further sonography reports. Visualization of one or more gestational sacs was defined as clinical pregnancy. Ongoing pregnancy was defined if pregnancy continued for more than 20 completed weeks of gestation. 

The patients were then followed until the end of the pregnancy.

***Statistical analysis***

Data was analyzed using SPSS software version 15. Chi-square, Student’s t-test and Fischer’s exact test were used to evaluate the differences between the groups. P value < 0.05 and confidence interval of 95% were considered to be statistically significant.

***Ethical Considerations***

Informed consent was obtained from all the participants.

## Results

**Figure 1:**
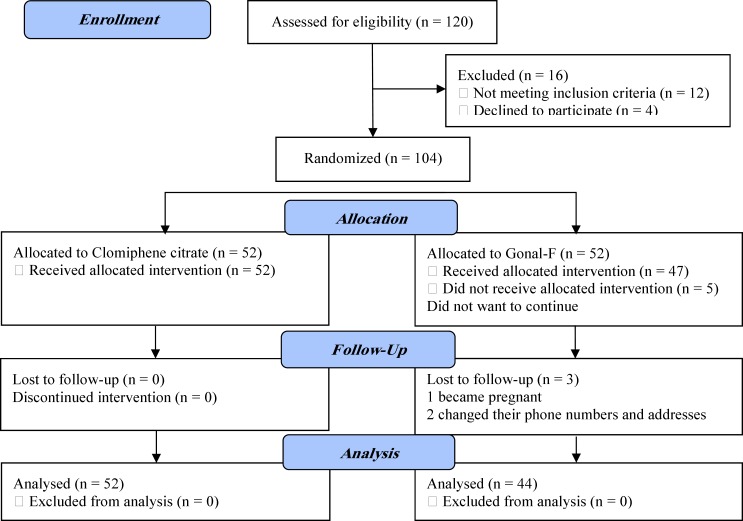
Flow Diagram

All the subjects in the CC arm completed the treatment course and entered the analysis. Of 52 women who were about to receive Gonal-F, 5 quitted the study due to personal reasons. Of the remaining 47, 2 women changed their addresses and phone numbers and one became pregnant in the middle. Therefore, 44 subjects in this arm finally entered the analysis. The consort flow chart is illustrated in figure 1. The demographic and clinical characteristics at the baseline were similar between the groups (Table 1).

As a whole, 11 clinical pregnancies (11.5%) were achieved. Table 2 shows the results per each trial group. In the CC group, 5 clinical pregnancies were achieved (9.6%) and all led to live births. Four of women who became pregnant in this group, 80%, were younger than 30 years old.

**Table 1:** Baseline demographic and clinical characteristics of the patients

	**All patients**	**Gonal-F**	**CC**	**p-value**
Number	96	52	44	
Age (years)	28.28 ± 4.71 a	27.52 ± 4.76	29.18 ± 4.52	0.084^b^
BMI (kg/m^2^)	25.09 ± 3.65	25.21 ± 3.54	24.95 ± 3.82	0.733
Infertility duration c	2.76 ± 1.69	2.57 ± 1.65	2.97 ± 1.73	0.283
Menstrual duration d	6.61 ± 6.24	6.15 ± 1.37	7.16 ± 9.13	0.435
Menstrual interval d	30.48 ± 8.86	31.06 ± 10.59	29.71 ± 5.83	0.438
Hormonal profile				
FSH e	5.67 ± 0.26	5.75 ± 0.36	5.57 ± 0.39	0.738
LH e	6.34 ± 0.52	6.80 ± 0.84	5.79 ± 0.55	0.339
PRL f	87.69 ± 15.29	79.83 ± 18.08	97.21 ± 25.92	0.574
AMH e	5.55 ± 0.56	5.19 ± 0.62	5.97 ± 0.98	0.494
Estradiol f	83.74 ± 11.51	70.32 ± 11.82	103.87 ± 22.27	0.155
Progesterone f	1.47 ± 0.14	1.63 ± 0.06	1.30 ± 0.26	0.289
DHEAS f	98.82 ± 12.16	85.90 ± 14.03	114.62 ± 20.79	0.242
Testosterone f	2.17 ± 0.87	1.94 ± 1.02	2.42 ± 1.44	0.782

a : mean ± SD;

b : student’s t test;

c : years;

d : days;

e : IU/L;

f : ng/dl

**Table 2:** Morphologic ultrasound features following ovulation induction

	**CC**	**Gonal-F**	**p-value**
Right ovary			
Dominant follicles a	1 ± 0.71 b	1.6 ± 1.17	0.004
Follicular size c	13.95 ± 8.13	14.55 ± 6.70	0.697
Left ovary			
Dominant follicles	1.49 ± 2.34	1.13 ± 0.98	0.363
Follicular size	13.49 ± 7.64	11.9 ± 8.32	0.339
Endometrial thickness (on the day of hCG injection)	7.95 ± 1.99	8.03 ± 2.05	0.851

a : number;

b : mean ± SD;

c : mm

In the Gonal-F group, 6 women became pregnant (13.6%) with 4 of them, 66%, younger than 30. Of the two pregnancies in those who were older than 30, one resulted in spontaneous abortion (2.3%). In the end, 5 successful deliveries were achieved in this group (11.4%).

The clinical pregnancy rate in the Gonal-F group and the CC group was 13.6% and 9.6% respectively and this difference was not significant (p = 0.538). The live birth rate was not significantly different between the groups either {11.4% versus 9.6%, p = 0.78} (Table 3).

According to our results, the mean number of dominant follicles of the right ovary was significantly higher after the treatment with FSH in comparison with CC, 1.60 ± 1.17 and 1 ± 0.71 respectively (p = 0.004). However, this difference was not significant for the left ovary (p = 0.363). The differences in mean follicular size after induction and the endometrial thickness on the day of hCG injection in two groups were not significant (Table 2). Neither OHSS nor multiple gestations were detected during the treatment cycle. In the Gonal-F group, the mean duration of stimulation was 8.65 ± 1.93 days with the minimum of 5 days and maximum of 15 days. Of the total of 46 patients in this group, the duration of stimulation was 8 days in 15 of them. The mean dose of Gonal-F prescribed was 430 ± 96 IU.

**Table 3:** Results comparing CC and Gonal-F

	**CC**	**Gonal-F**	**p-value**	**Odd’s ratio**	**Confidence interval (95%)**
Number of patients per protocol	52	44			
Clinical pregnancy	5 (9.6%)	6 (13.6%)	0.538	1.484	0.42-5.24
Ongoing pregnancy	5 (9.6%)	5 (11.4%)	0.780	1.205	0.325-4.68
Miscarriage	0	1 (2.3%)	0.274	1.203	0.978-4.68
Live birth rate	5 (9.6%)	5 (11.4%)	0.780	1.203	0.325-4.68
Cycle cancellation	0	0			
OHSS	0	0			

## Discussion

It has been more than 50 years that CC is established as the first-line agent for OI in women with PCOS. It is believed to be more convenient and cost-efficient (7, 19, 20).

Alternatives other than CC are aromatase inhibitors like letrozole which is considered to have more favorable ovarian and endometrial effects (3). Nonetheless, there are concerns regarding interruption with normal aromatase function in fetal tissues and hence the risk of birth defects with this oral agent (22). Other alternatives include insulin sensitizers and low dose gonadotropins. The application of low-dose gonadotropins for ovulation induction (OI) in infertile women with PCOS after failure of treatment with clomiphene citrate (CC) has become a common practice. PCOS constitutes 20-40% of the causes of female infertility (23); therefore, given the high prevalence of this disorder, first-line intervention for OI in this disorder is of paramount importance and a short term cost-effective treatment is demanded. There have been only two studies regarding the use of gonadotropins as the first-line strategy. In the first one published by Lopez et al (18), the cumulative pregnancy rate (CPR) in FSH group was comparable to those on CC treatment. In the second study by Homburg et al (19), however, the pregnancy rate (PR) was twice as high with low-dose recombinant FSH compared with CC. Furthermore, the cumulative clinical PR after 3 cycles was 52.1% for rFSH group compared with 41.2% for CC group. The cumulative LBR after 3 cycles was also higher for the FSH group (47.4% versus 36.9%). Although rFSH was associated with higher costs and less convenience, its use was justified as the first-line modality for ovulation induction in PCOS (19). According to our results, PR and LBR though apparently higher in the Gonal-F treatment group, were not different significantly (p > 0.05). The important point was nevertheless, the fact that in none of those who received Gonal-F, the cycle was cancelled and no case of OHSS happened. Moreover, of 6 pregnancies achieved in the Gonal-F group, all were singleton pregnancies. In the Homburg et al study, multiple pregnancy rate was 3.4%.

In the classic low dose regimen, the starting dose of 50-75 IU FSH is used for 7 to 14 days followed by weekly increment of 50% of the initial or the previous dose until the follicular development is initiated and this dosage is continued until the criteria for hCG administration is reached (15, 17, 21). According to Homburg study, what is critical in determining the mono ovulation and consequently better results is the amount of dosage increment and not the staring one (15, 21). Based on previous studies no significant difference was seen between the dosage of 37.5 and 50 IU as the starter (21). In his study with the starting dose of 50 IU FSH, successful results were obtained: mono-follicular ovulation in 83 % of the 69 cycles and pregnancy rate of 80 %. Only one cycle was cancelled and only one set of twin pregnancy developed (17).

Of course, the induction protocol can be varied according to the demographic features of different populations (21), as in another study in Vietnam, the starting dose of 25 IU FSH for a minimum of 14 days and an incremental dose of 25 IU when needed was regarded to be safe and resulted in 62.1% uni-follicular development and 15.8% cancellation rate (16). 

With the introduction of new Gonal-F pens in our country which are more user- friendly and make it feasible to have an increment of as low as 12.5 IU by a single click, the treatment protocol of our trial was designed. The starting dose was set to be 50 IU and the policy of increasing dose of 12.5 IU based on transvaginal sonographic monitoring was employed. This study presents the results of the first cycle of treatment only. Investigation on further cycles of treatment with more participants is under progress. Besides that, in continuation to the previous trials by Lopez and Homburg (18, 19), our goal was to further focus on recombinant FSH as the first-line agent for COI in PCOS women. We wanted to evaluate this presumption that instead of prescribing clomiphene first and then moving to FSH, by giving the FSH from the beginning we would reach better results in shorter time. The research hypothesis was that rFSH was better than CC in terms of efficiency. Contrary to our expectations, Gonal-F was not associated with superior outcomes and the research hypothesis was rejected. However, with smaller increments thanks to the newly designed pens, rFSH was safely used in PCOS patients even as their first treatment option without occurrence of OHSS or cancellation of the cycle.

Another point to be added is the better response of the right ovary compared to the left one following OI in the FSH group. The mean number of dominant follicles in the right ovary after induction with rFSH was significantly higher in comparison with CC. In other words, the right ovary responded better in the rFSH group. These findings were consistent with those of two other studies in which right ovarian responses were reported to be superior after stimulation with FSH compared with left ones (24, 25). Though histologically and embryologically similar, may be due to anatomical differences such as venous drainage, ovulation occurs more frequently in the right ovary (24).

## Conclusion

Recombinant FSH and clomiphene citrate had similar efficacy for the first cycle of OI in PCOS patients according to our results. 

The limitations of our study were the small number of patients evaluated and the assessment of only a single cycle of treatment. However, the safe usage of rFSH as the first-line treatment without occurrence of OHSS or the need for cancellation of the cycle was an invaluable positive aspect. Although it cannot be concluded from this study that recombinant FSH implication for the first cycle of OI in PCOS infertile women is superior to CC, considering the cumulative effects of gonadotropins, the authors believe that rFSH could be more efficacious than CC and yield in better results during 3-6 cycles of OI if more studies with larger study population be done.
